# Clinical Evaluation of Serum Tumor Markers in Patients With Advanced-Stage Non-Small Cell Lung Cancer Treated With Palliative Chemotherapy in China

**DOI:** 10.3389/fonc.2020.00800

**Published:** 2020-06-05

**Authors:** Muhammad Abbas, Said Abasse Kassim, Murad Habib, Xiaoyou Li, Meiqi Shi, Zhong-Chang Wang, Yiqiao Hu, Hai-Liang Zhu

**Affiliations:** ^1^State Key Laboratory of Pharmaceutical Biotechnology, Institute of Artificial Intelligence Biomedicine, Nanjing University, Nanjing, China; ^2^Department of Medical Oncology, Jiangsu Cancer Hospital and Jiangsu Institute of Cancer Research and Affiliated Cancer Hospital of Nanjing Medical University, Nanjing, China; ^3^Centre de Recherche en Gestion des Services de Sante, Faculté des Sciences de L'administration (FSA), Université Laval (UL), Centre Hospitalière Universitaire (CHU) de Québec UL-IUCPQ-UL, Québec, QC, Canada; ^4^Department of Surgery, Ayub Medical College, Abbottabad, Pakistan; ^5^Institute of Drug R&D, Medical School of Nanjing University, Nanjing, China

**Keywords:** non-small cell lung cancer, stage IV, serum tumor markers, prognosis, palliative chemotherapy, six-cycles

## Abstract

**Aim:** This study aims to analyze the prognostic value of seven tumor makers and also investigate the response of palliative chemotherapy in advanced NSCLC patients with advanced disease.

**Methods:** Medical records of 278 advanced NSCLC Chinese patients who received six cycles of palliative chemotherapy were retrospectively reviewed under ethical approval (JSCH2019K-011). Univariate and multivariate Cox regression analyses were performed using SPSS 24 to find the clinical value of these tumor markers and to identify the factors that were associated with progression-free survival (PFS), as well as the response to palliative chemotherapy.

**Results:** In baseline characteristic, the high levels of CEA, CA-125, CA-199, AFP, NSE, CYFRA21-1, and CA15-3 were detected in 209 (75.18%), 139 (50.0%), 62 (22.30%), 18 (6.47%), 155 (55.75%), 176 (63.30%), and 180 (64.74%) patients, respectively. Univariate analysis revealed that patients with high vs. normal levels of all tumor markers had an increased risk of poor prognosis. In the multivariable Cox regression model, the patient with (high vs. normal) CYFRA21-1 levels (HR = 1.454, *P* = 0.009) demonstrated an increased poor PFS. However, patients with (high vs. normal) CA19-9 levels (HR = 0.524, *P* < 0.0001) and NSE levels (HR = 0.584, *P* < 0.0001) presented a decreased risk of PFS. Also, patients receiving 3-drugs regimen had better PFS compared to those on 2-drugs regimen (*P* = 0.043).

**Conclusions:** The high levels of CYFRA21-1 was correlated with a poor prognostic factor of PFS for Advanced NSCLC patients. However, the high levels of CA19-9 and NSE were associated with a better prognostic factor of PFS. Additionally, smoking habits and tumor status had a poor prognostic factor of PFS. Moreover, we found that antiangiogenic therapy has high efficacy with first-line chemotherapy and longer PFS of NSCLC patients.

## Introduction

Lung cancer is one of the most common and fatal cancers worldwide (1, 2). Lung cancer is a heterogeneous disease comprising mainly non-small cell lung cancer (NSCLC) and small cell lung cancer (SCLC), which approximately accounted for 85% and 10–15% of all lung cancer cases respectively (2, 3). According to the global cancer statistics of 2018, lung cancer accounted for approximately 2,093,876 (11.6) new cases and 1,761,007 (18.4) of total cancer deaths representing one in five (18.4%) cancer deaths (4).

In China, the incidence and mortality rates of lung cancer have increased markedly over the past decades and accounted for ~49.94 per 100,000 men and 23.89 per 100,000 women and 40.30 per 100,000 and 17.13 per 100,000 deaths in 2014 (5). A recent study in China reported an annual mortality rate of 0.6 million (majority male) and 0.73 million new cases annually (6). In addition, NSCLC survival rate was estimated at 16.8% for men and 25.1% for women in 2012–2015, which are relatively low compared to other cancers (5). This can be explaining by the fact that about two-thirds of NSCLC patients are usually at an advanced stage (i.e., unresectable stage IIIB and IV) at the time of diagnosis (1, 2). Most of these advanced tumors are not surgically resectable as a result of disseminated (multiple sites) metastatic disease or metastatic sites that are not amenable to surgery. Patients with single metastatic sites may undergo surgical resection of both the primary tumor in the lung and the metastatic site. However, first-line chemotherapy used in most of the advanced NSCLC cases.

The purpose of palliative chemotherapy is to improve patient quality of life and increase the survival rate. Advanced non-small lung cancer patients are treated by either radiotherapy or palliative chemotherapy. Studies have reported that even with radiotherapy survival rates have not been significant (1, 2). Though palliative chemotherapy is not curative, it plays a supportive role to improve patient health state, and limit complications when chances of recovery are slim (7).

Tumor markers are small circulating quantifiable molecules present in blood or tissue which are released by tumor cells or body immune cells in response to tumor growth (8, 9). Tumor markers play a pivotal role in clinical diagnosis, prognosis, and anti-drug surveillance. Tumor markers can also be used to measure the response to chemotherapy (10, 11). Tumor markers have several advantages over conventional diagnostic methods, these are cheap, less time taking, unresting state, and avoid radiation exposure but statistically, it also supports the clinicians to estimate the progression of tumor (12, 13).

Previous studies have reported an association between tumor markers and curative effect in patients with breast cancer, epithelial ovarian cancer, gastric cancer, pancreatic cancer, and colorectal cancer (14–16). There is, however, limited clinical studies on the utilization of tumor markers in advanced-stage NSCLC (17). To the best of our knowledge, this is the first study to evaluate the clinical utility of seven tumor markers CEA, CA19-9, CA125, AFP, NSE, CA15-3, and CYFRA21-1 for prognostic specification as well as for measuring the response of chemotherapy (2-drugs vs. 3-drugs) in terminal stage (IV) NSCLC patients who underwent palliative chemotherapy.

## Methods

### Study Site

Jiangsu cancer hospital, also known as Jiangsu Institute of Cancer Research is founded in 1960 and located in Nanjing city, China. The Hospital has 1,161 open beds with 1,635 employees across 25 clinical and medical departments. In 2019, the medical oncology department of Hospital received over 4,874 patients, which present a monthly average of 406 patients.

### Study Design

**Figure 1:**
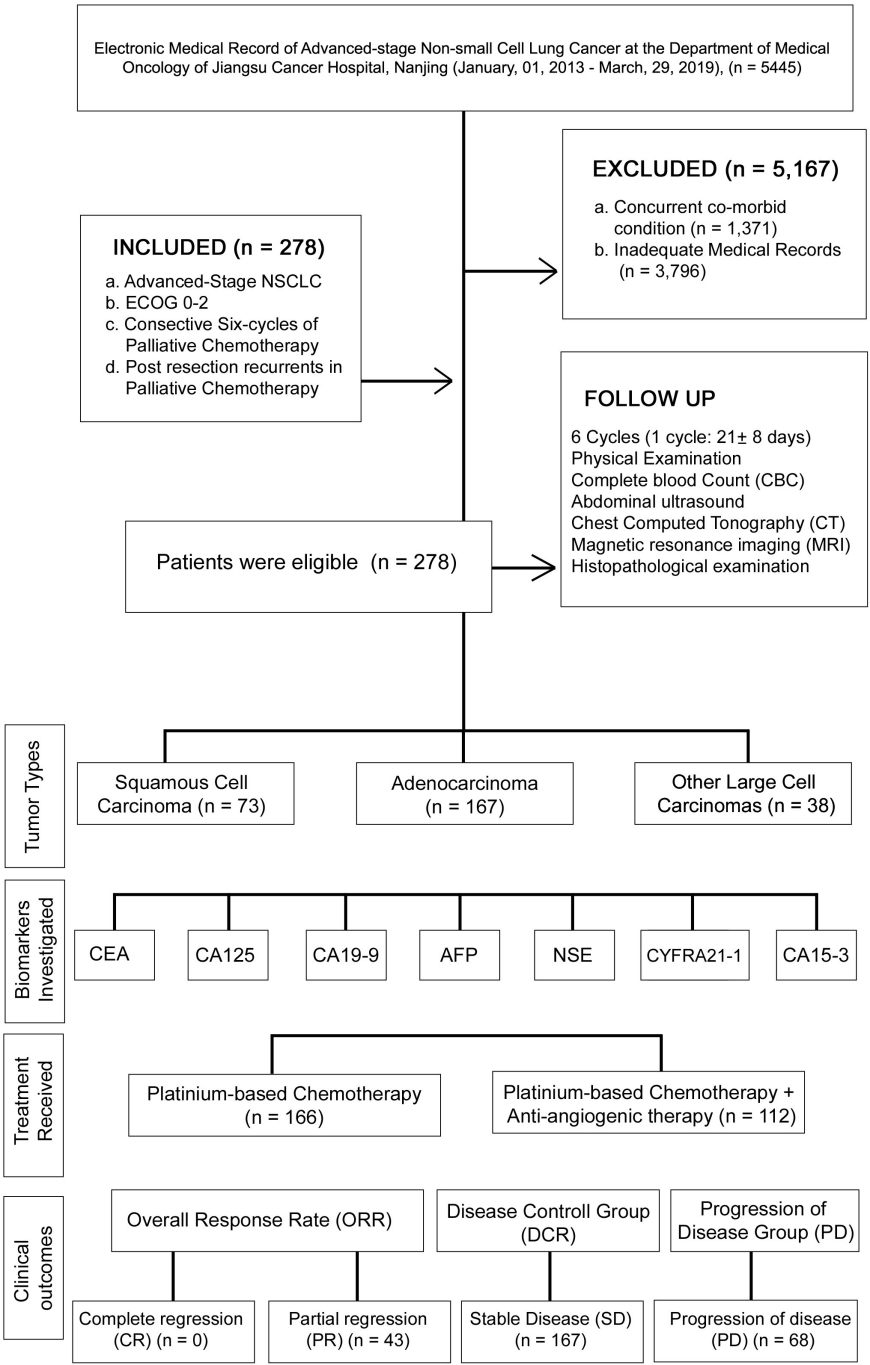
Procedural flowchart of the study.

A retrospective study was conducted between January 01, 2013, and March 29, 2019, under the approval of the research ethics committee of Jiangsu Cancer hospital (JSCH2019K-011). In this study, Medical records of 5,445 patients were succinctly reviewed and classified based on defined inclusion and exclusion criteria. The patient demographics, medical history, and physical examination, were verified before study entry. The patient medical record was collected until death, progression of cancer, and last medical fellow-up. The levels of CEA, CA125, CA19-9, AFP, NSE, CYFRA21-1, and CA15-3 were recorded at the baseline and at the start of six chemotherapy cycles. The flowchart and analysis are presented in Figure 1.

### Inclusion/Exclusion Criteria

The inclusion criteria of this study consisted of: (A) patients with histologically confirmed terminal stage IV NSCLC according to the TNM staging criteria set by the International Union Against Cancer (UICC) in 2009 (2); (B) patients with ECOG performance status of 0–2; (C) patients who received palliative chemotherapy and were followed up at least six chemo-cycles; and (D) Postresection recurrent of NSCLC patients in palliative chemotherapy. The exclusion criteria included: (A) patients who diagnosed previously or had concurrent co-morbid cancers; (B) patients with inadequate medical records or recurrence within six chemo-cycles. Based on the inclusion and exclusion criteria, a total of 278 advanced NSCLC patients were enrolled in this study.

### Laboratory Measurement

Assay of tumor markers was performed by electrochemiluminescence (ECL) so as to determine the baseline levels of CEA, CA19-9, CA125, AFP, CYFRA21-1, and NSE and at the beginning of each chemo-cycle until the risk of progression. The levels were compared with the manufacturer cutoff levels of: CEA < 3.5 ng/ml, CA125 < 35 U/ml, CA19-9 < 39 U/ml, AFP < 10 ng/ml, NSE < 16.3 ng/ml, CYFRA21-1 3.3 ng/ml and CA15-3 < 30 U/mL. Serum levels above (high) the cutoff values indicated a positive outcome. Positive detection of all the tumor markers was considered in case of one or more serum marker levels were above the normal cutoff range.

### Clinical Outcomes

The clinical outcomes were evaluated by progression free survival (PFS). PFS was an initial time of taking therapy to the tumor progression or death. The Curative response was measured by tomography accordingly to the Response Evaluation Criteria in Solid Tumor (RECIST) (1, 2). These were divided into complete regression (CR), stable disease (SD), partial response (PR), and progression disease (PD). Objective response rate (ORR) measured as CR and PR while SD considered as disease control rate (DCR).

### Treatment Received

All patients received palliative chemotherapy and were divided into two groups to assess the effectiveness of the chemotherapy: (1) patients receiving 2-drugs (Combination of chemotherapy) as indicated in Table 1 and (2) those receiving 3-drugs (Combination of chemotherapy + antiangiogenic therapy) as shown in Table 2.

**Table 1:** Palliative chemotherapy regimens for advanced-stage NSCLC patients.

**Combination of chemotherapy**	**Dose and cycle**
ETOPOSIDE + cisplatin	VP16 100 mg/m^2^, d1–3, Cis: 75 mg/m^2^, d1, q3w
PEMETREXED DISODIUM + carboplatin	Pem 500 mg/m^2^, d1, Carbo AUC 5, d1, q3w
PEMETREXED DISODIUM + irinotecan	Pem 500 mg/m^2^, d1, Iri 200 mg/m^2^, d1, q3w
DOCETAXEL + cisplatin	Doc 60–75 mg/m^2^, d1, Cis 60–75 mg/m^2^, d1
GEMCITABINE + vinorelbine	Gem 1,000 mg/m^2^ d1, d8, Vin 25 mg/m^2^ d1, d8, q3w
PACLITAXEL ALBUMIN + nedaplatin	Nab-Pac 125 mg/m^2^ d1, d8, Neda 80 mg/m^2^ d1, q3w
DOCETAXEL + epirubicin	Doc 60–75 mg/m^2^, d1, Epi 60 mg/m^2^, d1
BLEOMYCIN HCL + CARBOPLATIN	Bleo 15 mg, d1–5,Carbo AUC 5 d1, q3w
DOCETAXEL + oxaliplatin	Doc 60–75 mg/m^2^, d1, Oxol 120 mg/m^2^ d1, q3w
ETOPOSIDE + lobaplatin	VP16 100 mg/m^2*3^ (d1–3), Lobaplatin 30 mg/m^2^ d1, q3w
DISODIUM CANTHARIDINATE; PYRIDOXINE + pemetrexed	VP16 100 mg/m^2^, d1–3, Lobo 30 mg/m^2^, d1
PACLITAXEL ALBUMIN + cisplatin	Nab-Pac 125 mg/m^2^ d1, d8, Cis 60–75 mg/m^2^, q3w
PEMETREXED + tegafur; gimeracil; oteracil	Pem 500 mg/m^2^ d1, Tegafur 50 mg Bid*14, q3w
VINORELBINE TARTRATE + epirubicin	Vin 25 mg/m^2^ d1–3, Epi 60 mg/m^2^ d1, q3w

**Table 2:** Combination of chemotherapy plus anti-angiogenic agents' palliative chemotherapy-based regimens for advanced-stage NSCLC patients.

**Combination of chemotherapy plus anti-angiogenic agents**	**Dose and cycle**
PEMETREXED DISODIUM + carboplatin + bevacizumab	Pem 500 mg/m^2^, Carbo AUC 5 *1, Bev 7.5 mg/kg d1, q3w
DOCETAXEL + cisplatin + bevacizumab	Doc 60–75 mg/m^2^, d1, Cis 60–75 mg/m^2^ d1, Bev 7.5 mg/kg, d1, q3w
PEMETREXED DISODIUM + carboplatin + gefitinib	Pem 500 mg/m^2^, d1, Carbo AUC 5, d1, Gefi 250 mg/day, unti PD, q3w
PEMETREXED DISODIUM + carboplatin + osimertinib	Pem 500 mg/m^2^, d1, Carbo AUC 5, d1, Gefi 250 mg/day, unti PD, q3w
DOCETAXEL + oxaliplatin + icotinib	Doc 60–75 mg/m^2^, d1, Oxol 120 mg/m^2^ d1, Icotinib 125 mg tid until PD, q3w
Paclitaxel + carboplatin + bevacizumab	Pac 175 mg/m^2^, d1, Carbo AUC 5, d1, bev 7.5 mg/m^2^, d1, q3w
Gemcitabine + cisplatin + bevacizumab	Gem 1,000 mg/m^2^ d1, d8, Carbo AUC 5, d1, bev 7.5 mg/m^2^, d1, q3w

### Follow-Up

A standardized follow-up was received by all patients, for 2 years at an interval of 3 months, and 6 months, then 3 years and thereafter. On each cycle of follow-up, patients' physical examination, complete blood count (CBC), abdominal ultrasound, chest computed tomography (CT), and brain magnetic resonance imaging (MRI) were performed. Whenever possible local recurrence and distant metastases were also confirmed histologically.

### Statistical Analysis

All patients' medical record was analyzed using SPSS 24.0. The association between tumor markers and clinicopathological features were determined by Chi-square analysis. PFS distribution was estimated through Kaplan–Meier curves. The independent prognostic value of each tumor marker and clinicopathological features that highly affect the PFS was evaluated by Cox regression multivariate analysis. Change in the tumor marker levels and effectiveness of pre- and post-palliative chemotherapy were determined using Wilcoxon signed ranks test. And *P* < 0.05 was considered statistically significant.

## Results

### Patients' Characteristics

The Baseline characteristics of the 278 advanced NSCLC patients are summarized in Table 3. The Mean Age of the patients was (59.11 ± 10.39) years, and the majority of patients were males (65.8%) with no statistical differences (*P* = 0.357). In addition, 56.6% of patients had non-smoking habits with significant differences (*P* < 0.0001). Patients were classified according to the standard classification system of World Health Organization/International Association for the study of Lung Cancer (WHO/IASLC) (1, 2). With respect to the clinicopathological features, the majority of patients had metastasis (69.8%) with significant differences (*P* = 0.015). The histological diagnosis revealed 26.3, 60.1, and 13.7% of patients had squamous cell carcinoma, adenocarcinoma, and large cell carcinomas, respectively, with no significant differences (*P* = 0.152). Among these patients, there were 52.5% poorly differentiated, 15.1% moderate, and 32.4% well-differentiated. Most of the patients (59.7%) were on a 2-drugs regimen (Combination of chemotherapy), while the remaining (40.3%) received a 3-drugs regimen (Combination of chemotherapy plus antiangiogenic therapy). Of all patients, 75.9% presented a stable disease, while 24.5% had progression disease, with significant differences (*P* = 0.012).

**Table 3:** Baseline characteristics of tumor markers parameters of advanced-stage NSCLC patients.

**Variables**	**Patients *N* = 278 (%)**	***P*-value**	**CEA level**			**CEA125**			**CA19-9**			**AFP**			**NSE**			**CYFRA21-1**			**CA15-3**			**Combined detection**	
			**Normal**	**High**	***P*****-value**	**Normal**	**High**	***P*****-value**	**Normal**	**High**	***P-*****value**	**Normal**	**High**	***P-*****value**	**Normal**	**High**	***P-*****value**	**Normal**	**High**	***P-*****value**	**Normal**	**High**	***P-*****value**	**Combined** **+**	***P*****-value**
Age (Mean ± SD) years	(59.11 ± 10.39)	0.994			0.950			0.810			0.632			0.989			0.316			0.381			0.942		0.993
<60	124 (44.6)		31 (25.0)	93 (75.0)		61 (49.2)	63 (50.8)		98 (79.0)	26 (21.0)		116 (93.5)	8 (6.5)		59 (47.6)	65 (52.4)		49 (39.5)	75 (60.5)		44 (35.5)	80 (64.5)		124 (100)	
≥60	154 (55.4)		38 (24.7)	116 (75.3)		78 (50.6)	76 (49.4)		118 (76.6)	36 (23.4)		144 (93.5)	10 (6.5)		64 (41.6)	90 (58.4)		53 (34.4)	101 (65.6)		54 (35.1)	100 (64.9)		153 (99.3)	
Gender		0.357			0.678			0.528			0.955			0.555			0.451			0.017			0.893		0.352
Male	183 (65.8)		44 (24.0)	139 (76.0)		89 (48.6)	94 (51.4)		142 (77.6)	41 (22.4)		170 (92.9)	13 (7.1)		78 (42.6)	105 (57.4)		58 (31.7)	125 (68.3)		64 (35.0)	119 (65.0)		183 (100)	
Female	95 (34.2)		24 (26.3)	70 (73.7)		50 (52.6)	45 (47.4)		74 (77.9)	21 (22.1)		90 (94.7)	5 (5.3)		45 (47.4)	50 (52.6)		44 (46.3)	51 (53.7)		34 (35.8)	61 (64.2)		94 (99)	
Smoking status		<0.0001			0.784			0.503			0.149			0.033			0.391			0.112			0.520		0.0001
Non-smoker	157 (56.5)		42 (26.8)	115 (73.2)		82 (52.2)	75 (47.8)		127 (80.9)	30 (19.1)		151 (96.2)	6 (3.8)		68 (43.3)	89 (56.7)		640.84	93 (59.2)		59 (37.6)	98 (62.4)		156 (99.3)	
Smoker	88 (31.7)		15 (17.0)	73 (83.0)		40 (45.5)	48 (54.5)		65 (73.9)	23 (26.1)		80 (90.9)	8 (9.1)		35 (39.8)	53 (60.2)		28 (31.8)	60 (68.2)		26 (29.5)	62 (70.5)		88 (100)	
Unknown	33 (11.9)		12 (36.4)	21 (63.6)		17 (51.5)	16 (48.5)		24 (72.7)	9 (27.3)		29 (87.9)	4 (12.1)		20 (60.6)	13 (39.4)		10 (30.3)	23 (69.7)		13 (39.4)	20 (60.6)		33 (100)	
Metastasis		0.015			0.018			0.118			0.934			0.766			0.965			0.095			0.081		0.015
Yes	194 (69.8)		56 (28.9)	138 (71.1)		103 (53.1)	91 (46.9)		151 (77.8)	43 (22.2)		182 (93.8)	12 (6.2)		86 (44.3)	108 (55.7)		65 (33.5)	129 (66.5)		62 (32.0)	132 (68.0)		193 (99.48)	
No	84 (30.2)		13 (15.5)	71 (84.5)		36 (42.9)	48 (57.1)		65 (77.4)	19 (22.6)		78 (92.9)	6 (7.1)		37 (44.0)	47 (56.0)		37 (44.0)	47 (56.0)		36 (42.9)	48 (57.1)		84 (95.45)	
Differentiation		0.001			0.801			0.090			0.865			0.210			0.222			0.556			0.377		0.0001
Poor	146 (52.5)		36 (24.7)	110 (75.3)		79 (54.1)	67 (45.9)		113 (77.4)	33 (22.6)		139 (95.2)	7 (4.8)		59 (40.4)	87 (59.6)		52 (35.6)	94 (64.4)		55 (37.7)	91 (62.3)		145 (99.31)	
Moderate	42 (15.1)		9 (21.4)	33 (78.6)		22 (52.4)	20 (47.6)		35 (83.3)	7 (16.7)		39 (92.9)	3 (7.1)		21 (50.0)	21 (50.0)		14 (33.3)	28 (66.7)		14 (33.3)	28 (66.7)		42 (100)	
Unknown	90 (32.4)		24 (26.7)	66 (73.3)		38 (42.2)	52 (57.8)		68 (75.6)	22 (24.4)		82 (91.1)	8 (8.9)		43 (47.8)	47 (52.2)		36 (40.0)	54 (60.0)		29 (32.2)	61 (67.8)		90 (100)	
Tumor		0.152			0.213			0.063			0.670			0.890			0.786			0.088			0.413		0.155
Squamous cell	73 (26.3)		23 (31.5)	50 (68.5)		30 (41.1)	43 (58.9)		58 (79.5)	15 (20.5)		69 (94.5)	4 (5.5)		32 (43.8)	41 (56.2)		34 (46.6)	39 (53.4)		25 (34.2)	48 (65.5)		73 (100)	
Adenocarcinoma	167 (60.1)		36 (21.6)	130 (77.8)		87 (52.1)	80 (47.9)		129 (77.2)	38 (22.8)		155 (92.8)	12 (7.2)		73 (43.7)	94 (56.3)		55 (32.9)	112 (67.1)		56 (33.5)	111 (66.5)		166 (99.4)	
Other	38 (13.7)		9 (23.7)	29 (76.3)		22 (57.9)	16 (42.1)		29 (76.3)	9 (23.7)		36 (94.7)	2 (5.3)		18 (47.4)	20 (52.6)		13 (34.2)	25 (65.8)		17 (44.7)	21 (55.3)		38 (100)	
Drug		0.644			0.080			1.00			0.243			0.901			0.038			0.818			0.159		0.646
2-Drugs	166 (59.7)		35 (21.1)	131 (78.9)		83 (50.0)	83 (50.0)		125 (75.3)	41 (24.7)		155 (93.4)	11 (6.6)		65 (39.2)	101 (60.8)		60 (36.1)	106 (63.9)		53 (31.9)	113 (68.1)		166 (100)	
3-Drugs	112 (40.3)		34 (30.4)	78 (69.6)		56 (50)	56 (50)		91 (81.3)	21 (18.7)		105 (93.8)	7 (6.3)		58 (51.8)	54 (48.2)		42 (37.5)	70 (62.5)		45 (40.2)	67 (59.8)		111 (99.1)	
Response of therapy		0.012			0.969			0.164			0.343			0.366			0.592			0.045			0.082		0.012
CR (complete response)	0																								
PR + SD (stable disease)	210 (75.5)		52 (24.8)	158 (75.2)		110 (52.4)	100 (47.6)		166 (79)	44 (20)		198 (94.3)	12 (5.7)		91 (43.3)	119 (56.7)		84 (40.0)	126 (60.0)		80 (38.1)	130 (61.9)		209 (99.5)	
PD (progressive disease)	68 (24.5)		17 (25.0)	51 (75.0)		29 (42.6)	39 (57.4)		50 (73.5)	18 (26.5)		62 (91.2)	6 (8.8)		32 (47.1)	36 (52.9)		18 (26.5)	50 (73.5)		18 (26.5)	50 (73.5)		68 (100)	

### Association of Tumor Markers With Patients' Characteristics

In the pre-treatment, patients with high levels of CEA, CA-125, CA-199 AFP, NSE, CYFRA21-1, and CA15-3 were as follows: 209 (75.18%), 139 (50%), 62 (22.30%), 18 (6.47 %), 155 (55.75%), 176 (63.30%), and 180 (64.74%), respectively. In Table 3, CEA was found to significantly correlate with metastasis (*P* = 0.018). Similarly, CYFRA21-1 has strong correlation with gender (*P* = 0.017) and clinical response (*P* = 0.045). AFP correlated with smoking (*P* = 0.033) while NSE correlated only with therapy (*P* = 0.038). However, the combined positive detection of tumor markers was highly correlated with smoking (*P* = 0.0001), metastasis (*P* = 0.015) and cancer cell differentiation (*P* = 0.0001). There were no significant correlations in pre-treatment levels of CA125, CA-199, and CA15-3 levels with patients' characteristics (all *P* > 0.05), as shown in the Table 3.

**Figure 2:**
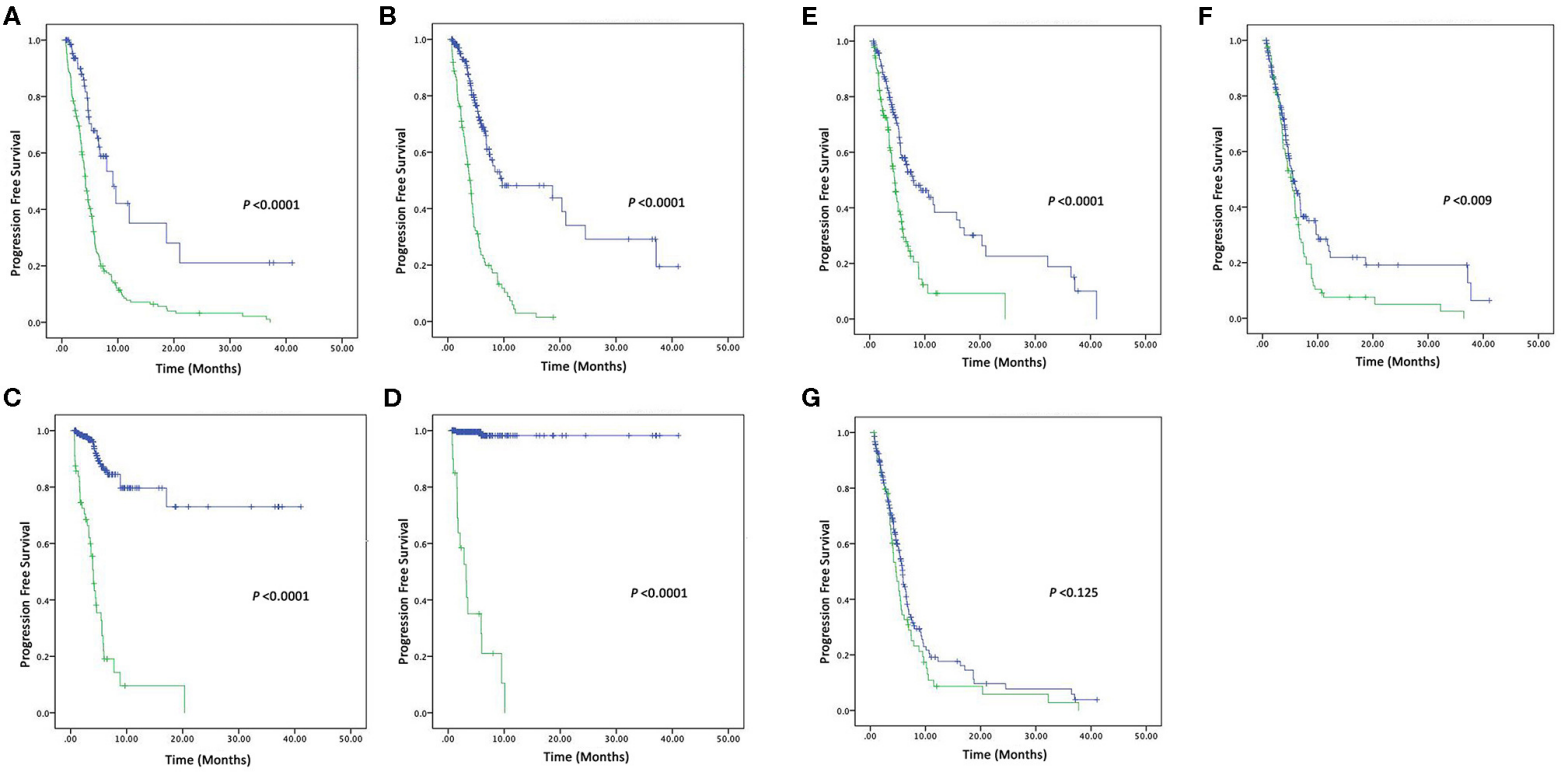
Kaplan–Meier survival curve of four Tumor markers **(A)** PFS of CEA (high vs. normal) levels, **(B)** PFS of CA125 (high vs. normal) levels, **(C)** PFS of CA19-9 (high vs. normal) levels, and **(D)** PFS of AFP (high vs. normal) levels. Kaplan–Meier survival curve of three Tumor markers **(E)** PFS of NSE (high vs. normal) levels, **(F)** PFS of CYFRA21-1 (high vs. normal) levels, and **(G)** PFS of CA15-3 (high vs. normal) levels.

In this present study, the tumor was progressed in 68 out of 278 patients, 166 patients used 2-drugs, while 112 patients used 3-drugs, and their overall median of PFS was 5.9 (4.1–8.7) months. Patients with CEA (high vs. normal) levels had a median PFS of 4.7 (4.15–5.31; *P* < 0.0001). Similarly, CA-125 (high vs. normal) levels median PFS was 6.26 (5.33–7.20; *P* < 0.0001) months. CA19-9 (high vs. normal) levels median PFS was 24.63 (20.41–28.85; *P* < 0.0001) months. AFP (high vs. normal) levels median PFS was 35.58 (32.40–38.76; *P* < 0.0001) months. NSE (high vs. normal) levels had median PFS was 5.6 (5.01–6.18; *P* < 0.0001) months. Similarly, patients with CYFRA21-1 (high vs. normal) levels median PFS was 5.4 (4.86–6.04; *P* = 0.009) months. However, patients with CA-153 (high vs. normal) levels were found poorly correlated with overall median PFS 5.53 (5.04–6.02; *P* = 0.125). Patients with elevated pre-treatment levels of CEA, CA125, CA19-9, AFP, NSE, CYFRA21-1, and CA15-3 noted shorter PFS compared to normal levels, as shown in the Figures 2A,G.

Furthermore, to find the pivotal role of these tumor markers as independent prognostic factors of PFS for NSCLC, univariate, and multivariate analyses were carried out, as shown in Tables 4, 5. Univariate Cox regression analysis was performed to assess the factors which correlated with PFS. CEA/CA125, and CA19-9 levels were found as highly associated with PFS. In addition, AFP and NSE levels were also statistical associated with PFS except in the following variables, i.e., Age > 60, Smoking status, Differentiation status, Tumor status, Therapy (3-drugs), and Curative response (disease progression).

**Table 4:** Univariate analysis of tumor markers for progression free survival using Cox regression model in advanced-stage NSCLC patients.

**Variables**	**CEA High vs. normal**		**CA125**		**CA19-9**		**AFP**		**NSE**		**CYFRA21-1**		**CA15-3**	
	**HR (95% CI)**	***P***	**HR (95% CI)**	***P***	**HR (95% CI)**	***P***	**HR (95% CI)**	***P***	**HR (95% CI)**	***P***	**HR (95% CI)**	***P***	**HR (95% CI)**	***P***
**Age**														
<60	2.662 (1.464–4.842)	0.001	3.321 (1.995–5.530)	<0.0001	11.013 (4.768–25.439)	<0.0001	97.792 (12.014–796.013)	<0.0001	2.887 (1.695–4.918)	<0.0001	1.547 (0.977–2.450)	0.063	1.034 (0.639–1.675)	0.891
>60	3.469 (1.904–6.318)	<0.0001	4.593 (2.814–7.499)	<0.0001	12.019 (5.879–24.572)	<0.0001	1.000 (0.075–13.399)	1.000	1.915 (1.247–2.940)	0.003	1.360 (0.917–2.017)	0.126	1.552 (1.006–2.393)	0.047
**Sex**														
Male	3.399 (1.917–6.027)	<0.0001	3.737 (2.427–5.756)	<0.0001	12.183 (6.146–24.153)	<0.0001	180.730 (23.400-−1395.894)	<0.0001	2.104 (1.413–3.133)	<0.0001	1.527 (1.073–2.175)	0.019	1.360 (0.923–2.004)	0.119
Female	2.594 (1.391–4.839)	0.003	4.579 (2.491–8.419)	<0.0001	10.286 (4.134–25.593)	<0.0001	65.309 (7.237–589.403)	<0.0001	2.600 (1.414–4.780)	0.002	1.294 (0.726–2.309)	0.382	1.077 (0.610–1.900)	0.799
**Smoking status**														
Non-smoker	2.567 (1.507–4.374)	0.001	4.939 (3.061–7.969)	<0.0001	11.319 (5.355–23.925)	<0.0001	46.447 (8.351–258.316)	<0.0001	2.941 (1.862–4.644)	<0.0001	1.289 (0.847–1.963)	0.236	1.419 (0.925–2.178)	0.109
Smoker	3.977 (1.889–8.36)	<0.0001	2.861 (1.527–5.360)	0.001	6.007 (2.606–13.845)	<0.0001	170554.306 (0.0001–1.509E+45)	0.797	1.622 (0.944–2.788)	0.080	1.684 (1.005–2.821)	0.048	1.275 (0.732–2.223)	0.391
Unknown	5.231 (0.692–39.598)	0.109	3.394 (1.231–9.361)	0.018	1129.174 (0.028–45709448.1)	0.194	11248.858 (0.0001–3.731E+21)	0.650	2.108 (0.593–7.497)	0.249	1.198 (0.495–2.900)	0.689	0.794 (0.299–2.111)	0.644
**Metastasis**														
Yes	3.368 (2.068–5.486)	<0.0001	6.252 (3.859–10.130)	<0.0001	13.335 (6.805–26.133)	<0.0001	1.000 (0.072–13.794)	1.000	2.304 (1.545–3.435)	<0.0001	1.378 (0.973–1.951)	0.071	1.107 (0.764–1.602)	0.592
No	1.956 (0.845–4.526)	0.117	2.007 (1.113–3.622)	0.021	8.953 (3.517–22.791)	<0.0001	35.267 (4.042–307.693)	0.001	2.150 (1.189–3.887)	0.011	1.887 (1.041–3.421)	0.037	1.905 (1.027–3.534)	0.041
**Differentiation**														
Poor	2.310 (1.375–3.883)	0.002	4.363 (2.546–7.479)	<0.0001	11.727 (5.406–25.441)	<0.0001	1.000 (0.020–50.669)	1.000	2.103 (1.344–3.291)	0.001	1.211 (0.800–1.832)	0.366	1.698 (1.058–2.726)	0.028
Moderate	4.243 (1.284–14.020)	0.018	3.920 (1.586–9.690)	0.003	31.435 (2.743–360.219)	0.006	161926.676 (0.0001–6.586E+68)	0.872	2.165 (0.829–5.658)	0.115	0.993 (0.457–2.158)	0.986	1.105 (0.477–2.557)	0.816
Unknown	4.202 (1.672–10.558)	0.002	3.575 (2.038–6.271)	<0.0001	12.361 (4.949–30.872)	<0.0001	63.787 (7.817–520.508)	<0.0001	2.493 (1.351–4.601)	0.003	2.376 (1.372–4.116)	0.002	1.183 (0.704–1.989)	0.526
**Tumor**														
Squamous	3.279 (1.288–8.347)	0.013	5.359 (2.798–10.265)	<0.0001	15.383 (4.874–48.554)	<0.0001	46.186 (4.718–452.122)	0.001	2.368 (1.246–4.500)	0.008	2.831 (1.480–5.414)	0.002	1.461 (0.811–2.634)	0.207
Adenocarcinoma	3.107 (1.810–5.332)	<0.0001	4.104 (2.548–6.610)	<0.0001	10.431 (5.245–20.748)	<0.0001	163.477 (20.983–1273.627)	<0.0001	2.478 (1.598–3.841)	<0.0001	1.069 (0.730–1.567)	0.731	1.461 (0.973–2.194)	0.067
Others	2.678 (1.016–7.060)	0.046	2.550 (0.914–7.116)	0.074	9.735 (2.566–36.943)	0.001	262777.899 (0.0001–3.364E+105)	0.915	1.547 (0.620–3.860)	0.350	1.118 (0.443–2.822)	0.814	0.861 (0.278–2.673)	0.796
**Drug**														
2–Drugs	2.483 (1.466–4.206)	0.001	3.843 (2.450–6.027)	<0.0001	9.267 (4.840–17.743)	<0.0001	57.450 (12.335–267.582)	<0.0001	2.004 (1.340–2.999)	0.001	1.666 (1.135–2.444)	0.009	1.230 (0.834–1.815)	0.297
3–Drugs	3.836 (1.912–7.697)	<0.0001	4.273 (2.426–7.527)	<0.0001	13.035 (5.122–33.172)	<0.0001	1.000 (0.034–29.436)	1.0000	2.667 (1.484–4.791)	0.001	1.262 (0.779–2.046)	0.344	1.256 (0.703–2.244)	0.441
**Curative response**														
CR	0													
PR + SD	2.773 (1.714–4.485)	<0.0001	3.459 (2.302–5.199)	<0.0001	13.619 (7.235–25.637)	<0.0001	69.883 (15.263–319.972)	<0.0001	2.114 (1.444–3.095)	<0.0001	1.616 (1.133–2.305)	0.008	1.545 (1.066–2.239)	0.022
PD	3.848 (1.632–9.074)	0.002	6.023 (2.831–12.817)	<0.0001	7.073 (2.539–19.704)	<0.0001	1.000 (0.032–31.230)	1.000	2.854 (1.434–5.681)	0.003	1.198 (0.682–2.104)	0.530	0.776 (0.400–1.507)	0.455

**Table 5:** Multivariate analysis of tumor markers for progression free survival using Cox regression model in advanced-stage NSCLC patients.

**Variables**	**HR**	**95%CI**	***P*****-value**
Age: <60 vs. >60	0.846	(0.655–1.092)	0.199
Sex: male vs. female	0.863	(0.612–1.218)	0.401
Smoking: ever vs. never	1.379	(1.020–1.864)	0.037
Unknown vs. never	1.1651	(0.786–1.727)	0.447
Treatment: 2- vs. 3-Drugs	1.183	(0.898–1.557)	0.231
Distant metastases: yes vs. no	0.954	(0.706–1.289)	0.758
Tumor: squamous vs. adenocarcinoma	0.650	(0.380–1.110)	0.114
Others vs. adenocarcinoma	4.030	(1.795–9.232)	0.001
Differentiation: moderate vs. poor	1.028	(0.709–1.492)	0.882
Unknown vs. poor	1.043	(0.730–1.492)	0.816
CEA: ≤ 3.5 vs. >3.5 ng/ml	0.851	(0.632–1.145)	0.286
CA125: ≤ 35 vs. >35 Uml	0.955	(0.724–1.261)	0.747
CA19-9: ≤ 39 vs. >39 U/ml	0.524	(0.375–0.731)	<0.0001
AFP: <10 vs. >10	0.672	(0.407–1.110)	0.121
NSE: ≤ 15.2 vs. >15.2 ng/ml	0.584	(0.446–0.763)	<0.0001
CYFRA21-1: <3.3 vs. >3.3	1.454	(1.098–1.926)	0.009
CA15-3: <30 vs. >30	1.310	(0.975–1.758)	0.073
Curative response: PR + SD vs. PD	0.886	(0.644–1.217)	0.454
Sex* Tumor (Squamous cells)	1.227	(0.671–2.244)	0.507
Sex* Tumor (Others)	0.336	(0.132–0.853)	0.022

In multivariable Cox regression model, smoking status (Ever vs. Never, *P* = 0.037), Tumor (Others vs. Adenocarcinoma, *P* = 0.001), CA19-9 (high vs. normal, *P* = < 0.0001) levels, NSE (high vs. normal, *P* = < 0.0001) levels, CYFRA21-1 (high vs. normal, *P* = 0.009) levels, CA15-3 (high vs. normal, *P* = 0.073) levels and Sex^*^ Tumor (*P* = 0.022) were found to be independent prognostic factors of PFS for NSCLC.

**Figure 3:**
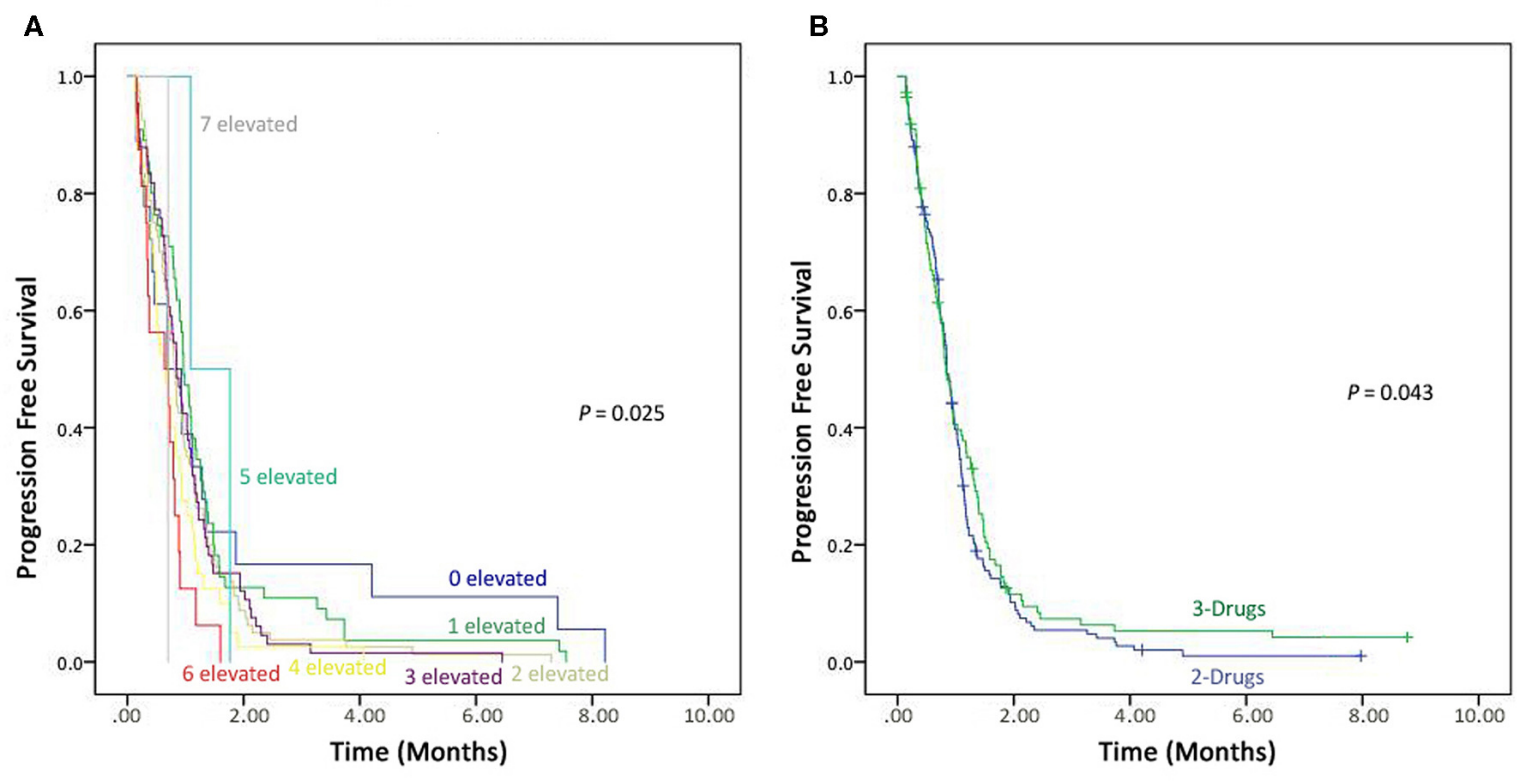
Kaplan–Meier progression-free survival. **(A)** Combined detection of elevated seven Tumor markers. **(B)** Comparison the effectiveness of 3-drugs regimen vs. 2-drugs regimen.

Prognostic values of all these tumor markers in advanced-stage NSCLC patients were evaluated in eight groups, i.e., (1) patients with one elevated tumor marker level, (2) patients with two elevated tumor markers levels, (3) patients with three elevated tumor markers levels, (4) patients with four elevated tumor markers levels, (5) patients with five elevated tumor markers levels, (6) patients with six elevated tumor marker levels, (7) patients with seven elevated tumor markers levels. However, only one patient found normal pre-treatment levels of all the seven tumor markers. On comparison of all the seven tumor markers, patients with six and seven were recorded shorter PFS compared to patients with normal pre-treatment levels (*P* = 0.025) as shown in the Figure 3A.

### Association of Treatment With Progression-Free Survival

In this study166 (59.7%) patients were on a 2-drug regimen, while 112 (40.3%) received a 3-drug treatment regimen. These therapies (2-drugs and 3-drugs) were compared for progression free survival, and those on the 3-drugs regimen found to have better PFS compared to the ones receiving the 2-drugs treatment regimen (*P* = 0.043), as shown in Figure 3B.

### Association of Tumor Markers With Response to Palliative Chemotherapy

In this study, 278 patients received palliative chemotherapy, and their clinical responses were recorded. None of the patients had fully recovered, while 43 patients achieved partial response (PR), 167 patients had stable disease (SD), and 68 patients had disease state progress (PD). Some patients had also experienced following side effects while receiving chemotherapy, i.e., alopecia, anorexia, nausea, vomiting. No patient death due to treatment was recorded.

Mean of the initial and final levels of the tumor markers were analyzed using Wilcoxon signed ranks test. The results revealed significant statistical Mean differences levels of CEA, CA-125, CA-199, AFP, and NSE (*P* = 0.019, 0.001, 0.023, *P* < 0.0001, and *P* = 0.012, respectively) between the pre-and post-treatment. Meanwhile, the Mean levels of CYFRA21-1 and CA15-3 were not statistically significant (*P* = 0.319 and 0.624, respectively), as shown in Table 6.

**Table 6:** Mean levels of seven tumor markers in pre- and post-palliative chemotherapy in advanced-stage NSCLC patients.

	**CEA Initial–CEA final**	**CA125 Initial–CA125 final**	**CA19-9 Initial–CA19-9 final**	**AFP Initial–AFP final**	**NSE Initial–NSE final**	**CYFRA21-1 Initial–CYFRA21-1 final**	**CA15-3 Initial–CA15-3 final**
*z* (Wilcoxon signed ranks test)^a^	−2.352^b^	−3.419^b^	−2.272^c^	−4.748^c^	−2.513^b^	−0.997^b^	−0.490^c^
*P*-value	0.019	0.001	0.023	<0.0001	0.012	0.319	0.624

a *Wilcoxon signed ranks test*.

b *Based on positive ranks*.

c *Based on negative ranks*.

All the seven tumor marker levels were measured at baseline and after 6th cycle of palliative chemotherapy. When stratified the Mean levels of all tumor markers by the disease control group and the progression disease group, there were statistical significant decreasing of CEA (*P* < 0.0001), CA-125 (*P* < 0.0001), AFP (*P* < 0.0001), NSE (*P* = 0.050), and CYFRA21-1 (*P* = 0.050) levels after the 6th cycle of palliative chemotherapy in the disease control group. However, no significant differences were observed in the Mean levels of pre- and post-treatment for CA19-9 (*P* = 0.151) and CA15-3 (*P* = 0.436) in the same group, as shown in Table 7.

**Table 7:** Mean levels of serum tumor markers in pre-and post-palliative chemotherapy in DC group (CR + PR + SD) and PD group respectively, in advanced-stage NSCLC patients.

**Efficacy comb**	**CEA initial–CEA final**	**CA125 Initial–CA125 final**	**CA19-9 Initial–CA19-9 final**	**AFP Initial–AFP final**	**NSE Initial–NSE final**	**CYFRA21-1 Initial–CYFRA21-1 final**	**CA15-3 Initial–CA15-3 final**
CR + SD + PR Z Asymp. Sig. (2-tailed)^a^	−3.517^b^	−4.559^b^	−1.435^c^	−4.476^c^	−1.897^b^	−1.958^b^	−0.779^c^
	0.000	0.000	0.151	0.000	0.050	0.050	0.436
PD Z Asymp. Sig. (2-tailed)^a^	−1.265^c^	−0.956^c^	−1.983^c^	−1.725^c^	−1.752^b^	−0.947^c^	−0.633^b^
	0.206	0.339	0.047	0.084	0.080	0.344	0.527

a *Wilcoxon signed ranks test*.

b *Based on positive ranks*.

c *Based on negative ranks*.

In addition, when stratified by the progression disease group, there was statistical significant decrease of CA19-9 (*P* = 0.047) levels between the pre-and post-treatment. However, no significant differences were observed for CEA, CA125, AFP, NSE, CYFRA21-1, and CA15-3 levels in the progression disease group (all *P* > 0.05), as shown in Table 7.

Furthermore, we also evaluated the response to therapy in patients receiving the two forms of palliative chemotherapy (i.e., 2-drugs or 3-drugs regiment). As evinced from Table 7, patients receiving a 3-drugs treatment regimen achieved better therapeutic outcomes compare to those on a 2-drugs regimen. Also, the pre- and post-treatment levels of the tumor markers were compared. When stratified by 3-drugs regimen, the results showed significant differences in CA125 (*P* = 0.009), AFP(*P* < 0.0001), NSE (*P* = 0.014) and CYFRA21-1 (*P* = 0.43) levels. However, no significant differences were observed for CEA (*P* = 0.122), CA19-9 (*P* = 0.071), and CA15-3 (*P* = 0.983) levels. Meanwhile, when stratified by the 2-drugs regimen, no statistical significant differences were observed in all tumor markers (all *P* > 0.05), as shown in Table 8.

**Table 8:** Comparing the clinical response of palliative chemotherapy (3-Drugs and 2-Drugs) in advanced-stage NSCLC patients.

**Efficacy**	**CEA Initial–CEA final**	**CA125 Initial–CA125 final**	**CA19-9 Initial–CA19-9 final**	**AFP Initial–AFP final**	**NSE Initial–NSE final**	**CYFRA21-1 Initial–CYFRA21-1 final**	**CA15-3 Initial–CA15-3 final**
3-Drugs Z Asymp. Sig. (2-tailed)^a^	−1.546^b^	−2.622^b^	−1.805^c^	−4.807^c^	−2.468^b^	−2.021^b^	−0.021^b^
	0.122	0.009	0.071	0.000	0.014	0.043	0.983
2-Drugs Z Asymp. Sig. (2-tailed)^a^	−1.839^b^	−2.170^b^	−1.424^c^	−1.365^c^	−0.887^b^	−0.866^c^	−0.731^c^
	0.066	0.30	0.154	0.172	0.375	0.386	0.465

a *Wilcoxon signed ranks test*.

b *Based on positive ranks*.

c *Based on negative ranks*.

## Discussion

This retrospective study is one of the few studies that assess the clinical utility of tumor markers CEA, CA19-9, CA125, AFP, NSE, CA15-3, and CYFRA21-1 for prognostic specification as well as for measuring the response to chemotherapy. CEA is non-specific with an abnormal countenance in solid tumors including, non-small lung cancer. Moro et al. (18) reported CEA as a negative prognostic factor. One study reported that CEA has a poor prognostic specification in NSCLC for survival (19). In our present study, patients having elevated CEA pre-treatment levels were correlated with shorter PFS and poor prognosis compared to those with normal levels, as similarly found in previous studies (19, 20). Moreover, in univariate Cox regression analysis, CEA was a correlated factor with PFS, but the multivariate analysis demonstrated that CEA is not an independent prognostic factors of PFS (*P* = 0.286).

Previously, the role of CA125 as a prognostic marker was not well defined (21). A limited number of studies had explored its prognostic value in an advanced-stage of cancer (22, 23). Herein, patients with increased pre-treatment levels of CA125 had not shown any significance, but in univariate Cox regression, CA125 was found statistical associated with risk of progression. But the multivariate analysis found no statistical significant (*P* = 0.747). Similarly, the role of CA19-9 was not previously well-elucidated with PFS in NSCLC patients (19, 24). However, in our study patients with increased pre-treatment levels of CA19-9 had not shown any significant differences (P > 0.05), but in univariate Cox regression and multivariate variable models, CA19-9 was found as an independent prognostic factor associated with risk of progression.

The prognostic value of AFP is already reported in several types of cancers (e.g., gastric cancer and ovarian cancer) (25), but there is no study available that explored its diagnostic and prognostic value in lung cancer (26). Our study is the first to our best knowledge to identify the potential role of AFP in NSCLC. Our results showed that AFP levels have a significance difference in high pre-treatment levels. Moreover, AFP was found associated with PFS in univariate Cox regression, but not in multivariate analysis (*P* = 0.121). Further studies are, however needed to validate our results.

The role of NSE as a tumor marker is widely accepted in small cell lung cancer (SCLC). However, its prognostic value is controversial in NSCLC (27). Numerous studies explored the prognostic role of NSE in local advanced and metastatic NSCLC and found it as a vital prognostic factor for PFS. (28, 29) Our findings are also consistent with them and found NSE levels were associated with worse prognosis and shorter PFS. On the contrary, one study on 67 operable early stage NSCLC patients reported a non-correlation of NSE with prognosis (30). In addition, studies explored the prognostic reliability of CYFRA21-1 and its levels were highly expressed the in blood of NSCLC (31). In alignment with our study, we found a significant correlation of CYFRA21-1 with gender and curative response. Furthermore, univariate cox regression and multivariate variable model results showed that CYFRA21-1 is a reliable tumor marker of NSCLC. Our findings are also in line with previous studies that found CYFRA21-1 as an independent predictor of gender and metastasis (32).

CA15-3 is a mucin-1 soluble form that is associated with non-squamous carcinoma (33). We did not find any significant difference in pre-treatment levels of CA15-3. However, univariate Cox regression revealed that CA15-3 was associated with Age, poor differentiation, and disease control group, but no significant differences were observed in multivariate analysis. In accordance with our findings, Liu et al. (34) reported that CA15-3 is not a reliable tumor marker. Furthermore, CEA, CA125, CA19-9, AFP, NSE, CYFRA21-1, and CA15-3 may not have significant prognostic values individually, but their combined detection can help in diagnosis, prognosis, and further, it can also evaluate the response of therapy. One study reported that changes in tumor marker levels in patients taking pre- and post-gefitinib-based chemotherapy were associated with tumor response and PFS (35). Therefore, the clinical utilization of these tumor markers could play a promising role in predicting the outcomes of therapy in NSCLC. The combined positive detection was highly correlated with smoking status, metastasis, differentiation, and curative response.

In the Kaplan–Meier survival curve, patients with 5-, 6-, or 7-elevated pre-treatment tumor markers have short PFS compared to those with 0, 1-, 2-, 3-, or 4-elevated pre-treatment tumor markers. Therefore, clinicians/oncologists should consider the detection of the combined tumor markers before prescribing the chemotherapy (36–38). The role of chemotherapy in advanced-stage NSCLC in the past two decades has been well-established. However, an antiangiogenic drug also gained attention in recent years, antiangiogenic drugs, e.g., bevacizumab has proved its efficacy in numerous solid tumors, and also show high efficacy with first-line chemotherapy in NSCLC patients (39, 40). Numerous studies reported the safety profile and synergistic effects of bevacizumab in combination with chemotherapy (40, 41). Herein, patients who received 3-drugs regimen had longer PFS compared to those on 2-drugs. Those findings were consistent with previous studies (42).

The association between tumor markers and curative effect has already been studied in breast cancer, colorectal cancer, gastric cancer, pancreatic cancer, and ovarian cancer, but limited clinical studies are available to identify the role of tumor markers and response to chemotherapy in advanced stage of NSCLC (14, 16, 43–45). In this study, we sought to determine the clinical potential of tumor markers in monitoring the response of patients to palliative chemotherapy. Our results showed a significant reduction of tumor marker levels after palliative chemotherapy, especially in the disease control group (CR + PR + SD), as compared to the progression disease group, as aforementioned in Table 7.

In the present study, we also compared the effectiveness of a 2- and 3-drugs combination therapy. Our results showed significant differences in the tumor marker levels of patients using 3-drugs than those on a 2-drugs therapy, as shown in Table 8. Previously published studies supported the hypothesis that antiangiogenic therapy, e.g., bevacizumab, can penetrate inside the tumor with or without first line chemotherapy (1, 2). It can therefore be inferred that combination of antiangiogenic therapy with chemotherapy could improve patient survival and improve their quality of life.

The limitation of this retrospective study is that the socio-demographic data may be subject to bias, especially for the classification of being smoker, considering the fact it was a self-report. Nonetheless, our findings require confirmation in additional large prospective studies.

## Conclusion

The high levels of CYFRA21-1 were correlated with poor a prognostic factor of PFS for Advanced NSCLC patients. However, the high levels of CA19-9 and NSE were associated with a better prognostic factor of PFS. Additionally, smoking habits and tumor status had a poor prognostic factor of PFS. Moreover, we found that antiangiogenic therapy has high efficacy with combination of chemotherapy and longer PFS of NSCLC patients.

## Data Availability Statement

All datasets generated for this study are included in the article/supplementary material.

## Ethics Statement

The studies involving human participants were reviewed and approved by the ethics research committee of Jiangsu Cancer hospital, Nanjing, China. The patients/participants provided their written informed consent to participate in this study.

## Author Contributions

MA and MS: conceptualization. MA: methodology and writing—original draft preparation. MA and SK: formal analysis. MA and XL: data curation. MA, SK, and MH: writing—review and editing. Z-CW, MS, YH, and H-LZ: supervision and project administration.

## Conflict of Interest

The authors declare that the research was conducted in the absence of any commercial or financial relationships that could be construed as a potential conflict of interest.
